# Supplementary material for: Perceptions of people with Parkinson’s and their caregivers of falling and falls-related healthcare services- a qualitative study
Source: PLoS One. 2022 Oct 26;17(10):e0276588. doi: 10.1371/journal.pone.0276588 (PMC9604942; doi:10.1371/journal.pone.0276588)
Supplement: S1 File — Semi-structured interview schedule for PwPD and caregivers. (DOCX) [file pone.0276588.s001.docx]

**Interview schedule**

To start off I would like to ask a few questions about falls.

1. Tell me about your experience of falling

| **Probes** | **Prompts** |
| --- | --- |
| Tell me about your most recent fall  Are all your falls similar or very different to one another? | Can you tell me about any times when you stumble but manage to stop yourself from falling?  Can you tell me about the preventable or unpreventable nature of your falls? |
|  |  |

1. Can you describe whether your falling has changed how you do your day to day activities?

| **Probes** | **Prompts** |
| --- | --- |
| Can you tell me about…   1. Anything that you now do differently to reduce your risk of falling? 2. Anything that you have found particularly helpful? 3. Anything that you have found particularly unhelpful? |  |
|  |  |

1. How does falling make you feel?

| **Probes** | **Prompts** |
| --- | --- |
| Can you explain about whether these feelings have affected how you perform your day to day activities? | Can you tell me about any concerns that you might have?   1. Anything that you now do differently as a result of these concerns? |

I would now like to ask you a few questions about your understanding of falling and of sources of information that are available to help you.

1. Can you tell me about which healthcare professionals, if any, you have told about your falls?

We would like to know the professions of those that you have told, not the names of individuals

| **Probes** | **Prompts** |
| --- | --- |
| What role do you think that these professionals might play in helping with falls? | Can you tell me about the role that you think that…   1. Your Parkinson’s specialist nurse might play in helping with falls? 2. Your Parkinson’s specialist doctor might play in helping with falls? |
|  |  |

1. Where, if anywhere, have you found information that might help you to:
   1. Reduce your risk of falling?
   2. Reduce the risk of injury?
   3. To cope after a fall?

| **Probes** | **Prompts** |
| --- | --- |
| Can you tell me about your experience of these?  Can you explain to me whether you find these useful? | Can you explain any support that you might have had through your P (UK) support group for falling?  Are you aware of the leaflet available through P (UK)? If so…   1. How do you find this? 2. How do you find this to read? 3. How do you find this to navigate?   Can you tell me about any other sources that are available such as those on the internet or on YouTube |

1. Can you tell me about anything relating to falling that you have learnt ‘along the way’?

| **Probes** | **Prompts** |
| --- | --- |
| Can you explain how this might have altered your   1. Risk of falling? 2. Risk of injury?   Can you explain how this might have altered any concerns that you might have in relation to falling? |  |
|  |  |

1. Can you tell me about whether you think there is any information that you need that might help you to…
   1. Reduce your risk of falling?
   2. Reduce the risk of injury?
   3. To cope after a fall?

| **Probes** | **Prompts** |
| --- | --- |
| What would be a good or an appropriate way for you to get this information? |  |
|  |  |

We are looking to design a guide to help people with Parkinson’s who fall, and those close to them*

1. Can you tell me about what you think should be included in our guide?*

| Probes | Prompts |
| --- | --- |
| Can you explain how you think this information should be provided?  Can you explain what you think our guide should look like? |  |
|  |  |

1. Can you explain any further suggestions that you might have to help us to develop our guide?*

** N.B. This study was part of a programme of research. Following on from this study we sought to design a guide to support people with Parkinson’s disease and their caregivers.*
