# Supplementary material for: Perceptions of people with Parkinson’s and their caregivers of falling and falls-related healthcare services- a qualitative study
Source: PLoS One. 2022 Oct 26;17(10):e0276588. doi: 10.1371/journal.pone.0276588 (PMC9604942; doi:10.1371/journal.pone.0276588)
Supplement: Attachment — Submitted filename: Response to reviewers.docx [file pone.0276588.s002.docx]

**Response to reviewers**

Response to reviewer feedback is outlined in the table below. Line references are those within the marked up copy of the manuscript.

| Reviewer Comment | Change made |
| --- | --- |
| line 87: spell out HCP as first time use of abbreviation | Line 87: Thank you for highlighting this omission. We have now spelt out ‘healthcare professionals’ on first use of the abbreviation ‘HCPs’ |
| line 90:  possible change “in the setting” to “context” | Thank you for highlighting this. We have made this suggested change on line 90. |
| Methods |  |
| Your paper needs to  describe in more detail with justification, the methodology you used to address your research questions and have a section on research team reflexivity | Thank you for prompting us to provide a more thorough description, with justification, of the methodology used in our research. We have addressed this and included more details of our study design and methodology decisions under the subheading ‘Study Design’ on lines 120-133. Thank you also for highlighting the importance of including a section on research team reflexivity, which we have addressed through the addition of the subsection titled ‘researcher backgrounds and reflexivity’ on lines 211-228. |
| Who developed and how (based on what) the interview questions. I think the key questions should be in the body of the paper (not in a supplementary file) as they inform the nature of the data collected | Thank you for highlighting that we have not described who, and how, we developed our interview questions. Our questions were developed by CO, LD and HCR, as is now described within the text (lines 162-167). The interview schedule underwent minor changes during data collection as we have now described. As suggested, we have now inserted a figure which details the key interview questions (Figure 1, line 168). |
| What experience or training did the interviewers have in qualitative interviewing? | Thank you for prompting us to describe the training that CO and CG received in qualitative interviewing. We have addressed this by describing the training and supervision that CO and CG received on lines 177-180. |
| When were field notes collected and what sort of field notes were collected? | Thank you for highlighting that we have not described our field notes in detail. We have detailed the field notes that we collected, and how this aided our analysis, on lines 180-185. |
| Far more detail of what methodological approach you used in your data analysis and a more detailed description of what you actually did in this analysis. | Thank you for highlighting that we needed to provide more detail about our methodological approach. We have addressed this by providing additional details to include a description of the subtype of thematic analysis that we used, how we coded our data and how we developed out subthemes and themes (lines 186-210). |
| Line 161-162: why after 18 interviews had been coded ... and not 19 or 20 or ... | Thank you for highlighting that we had not described why we chose this timepoint. We chose this timepoint as during discussions within the research team it became clear that the number of codes was becoming unwieldly, and that many of the current codes could be grouped to support the analysis of the data. This is now described within the manuscript (lines 201-205). |
| In gaining consent from caregivers for those participants who had cognitive impairment/dementia, what did you do to affirm that the person agreed with their involvement - did you check with them in some way? | Thank you for highlighting this important ethical consideration. We asked both PwPD and their caregivers whether they wanted to participate. Consultee declaration forms were completed when the PwPD wished to participate but lacked the capacity to provide informed written consent. We have added these additional details to the manuscript (lines 231-233). |
| Results |  |
| You should not start a sentence with a number, it should be spell out. | Thank you for raising this grammatical error. Due to the changes that we have made in response to the reviewer comment below, the sentence no longer starts with ‘38’ |
| Please state how many people volunteered, how many were ineligible etc. | Thank you for highlighting that we had not provided details of the number of people who were eligible to take part in the study. We have addressed this by detailing the number of questionnaires distributed and returned. All PwPD invited to interview using the purposive sampling technique agreed to participate. (lines 147-158). We have added these details into the methods section to eliminate overlap between the methods and results sections. |
| Other minor typographical or presentation issues have been corrected on line 236 (addition of ‘participant characteristics’ subheading), line 252 (altered ‘findings’ to ‘themes’ to match the descriptor used in the main body of the manuscript) and line 255 (started each theme name with a capital letter). | |

Journal requirements

Response to the feedback that we received about the journal requirements is detailed below.

| Journal requirements | Changes made |
| --- | --- |
| Please ensure that your manuscript meets PLOS ONE's style requirements, including those for file naming. The PLOS ONE style templates can be found at | Thank you for this feedback. We have re-read the style requirements in each of the PDFs quoted and are unable to identify any areas where our manuscript does not meet these. Correspondence from yourselves on 24^th^ June 2022 advised that this text is included in all revision decision letters to ensure that PLOS style is adhered to. We feel that our manuscript is in line with PLOS style guidelines and have therefore not made any additional edits here. Please advise us if you do identify any changes that we need to make and we will implement these. |
| We note that the grant information you provided in the ‘Funding Information’ and ‘Financial Disclosure’ sections do not match.  When you resubmit, please ensure that you provide the correct grant numbers for the awards you received for your study in the ‘Funding Information’ section. | Dr Charlotte Owen was supported by the University of Southampton NIHR Academic Clinical Fellow (ACF) training programme (<https://www.nihr.ac.uk/funding/nihr-academic-clinical-fellowships-in-medicine-2021/25719>), and subsequently went on to be awarded a fellowship by the National Institute for Health Research Applied Research Collaboration (NIHR ARC) Wessex. Work completed during the ACF training programme, and the fellowship formed the research for this manuscript. No grants were awarded for this research, we are sorry that this was unclear from the information provided. In the ‘funding information’ section within the submission system we have still listed Dr Owen’s NIHR ARC fellowship, please advise us if you would like for us to remove the fellowship listing from this section to alleviate any confusion. |
| We note that you have indicated that data from this study are available upon request. PLOS only allows data to be available upon request if there are legal or ethical restrictions on sharing data publicly. For more information on unacceptable data access restrictions, please see <http://journals.plos.org/plosone/s/data-availability#loc-unacceptable-data-access-restrictions>.  In your revised cover letter, please address the following prompts:  a) If there are ethical or legal restrictions on sharing a de-identified data set, please explain them in detail (e.g., data contain potentially sensitive information, data are owned by a third-party organization, etc.) and who has imposed them (e.g., an ethics committee). Please also provide contact information for a data access committee, ethics committee, or other institutional body to which data requests may be sent. | There are ethical restrictions on sharing our de-identified data set, as manuscripts contain potentially sensitive information, as imposed by the University of Southampton Faculty of Medicine ethics committee ([ergo@soton.ac.uk](mailto:ergo@soton.ac.uk), reference 29763). Bona fide researchers, subject to registration may request supporting data via University of Southampton repository <https://doi.org/10.5258/SOTON/D2329>.  We have provided this information on our cover letter alongside their contact details. |
| Please note that in order to use the direct billing option the corresponding author must be affiliated with the chosen institute. Please either amend your manuscript to change the affiliation or corresponding author, or email us at [plosone@plos.org](mailto:plosone@plos.org) with a request to remove this option. | Thank you for highlighting that the corresponding author is not currently employed by the University of Southampton. We have amended the corresponding author (line 6) to Dr Laura Dennison, who is employed by the University of Southampton, and provided her email address on line 13. |
| Please review your reference list to ensure that it is complete and correct. If you have cited papers that have been retracted, please include the rationale for doing so in the manuscript text, or remove these references and replace them with relevant current references. Any changes to the reference list should be mentioned in the rebuttal letter that accompanies your revised manuscript. If you need to cite a retracted article, indicate the article’s retracted status in the References list and also include a citation and full reference for the retraction notice | Thank you for this feedback. We have reviewed all of our references and we cannot locate a reference that has been retracted. Correspondence from yourselves on 24^th^ June 2022 advised that this text is included in all revision decision letters to ensure that PLOS style is adhered to. On reviewing our references we did identify that references 46 and 64 were the same publication (McLaughlin et al.). To rectify this we have removed reference 64 and references after reference 64 have consequently been renumbered. We have also edited our referencing style to adhere to PLOS style; citation numbers in the manuscript are now within square and not round backets. |
